# Development of the Nanobeam X-ray Experiments instrument at PAL-XFEL

**DOI:** 10.1107/S1600577525000426

**Published:** 2025-02-12

**Authors:** Jangwoo Kim, HyoJung Hyun, Seonghan Kim, Sun Min Hwang, Myong-Jin Kim, Dogeun Jang, Kyung Sook Kim, Jaeyong Shin, Sejin Kim, Junha Hwang, Sung Yun Lee, Eunyoung Park, Sangsoo Kim, Intae Eom, Changyong Song, Daewoong Nam

**Affiliations:** a XFEL Beamline Department, Pohang Accelerator Laboratory Pohang University of Science and Technology Pohang37673 Republic of Korea; b PLS-II Beamline Department, Pohang Accelerator Laboratory Pohang University of Science and Technology Pohang37673 Republic of Korea; c Photon Science Center Pohang University of Science and Technology Pohang37673 Republic of Korea; d 4GSR Research Division, Pohang Accelerator Laboratory Pohang University of Science and Technology Pohang37673 Republic of Korea; e Department of Physics Pohang University of Science and Technology Pohang37673 Republic of Korea; f Center for Ultrafast Science in Quantum Matter, Max Planck POSTECH/Korea Research Initiative, Pohang37673, Republic of Korea; ESRF – The European Synchrotron, France

**Keywords:** XFELs, X-ray free electron lasers, PAL-XFEL, nanofocusing, coherent diffraction imaging

## Abstract

A Nanobeam X-ray Experiments (NXE) instrument was developed and installed at the hard X-ray beamline of the Pohang Accelerator Laboratory X-ray Free Electron Laser. This instrument consists of a diagnostic system, focusing optics, an X-ray diffraction endstation and a femtosecond laser delivery system. The NXE instrument enables sophisticated X-ray experiments using nanofocused X-rays. At a 9.5 keV X-ray energy, the beam was successfully focused to 390 nm × 230 nm at the focal plane using Kirkpatrick–Baez mirrors. Following the successful commissioning experiments in December 2021 and April 2022, the instrument became available for regular user experiments in January 2023. The first user experiment was conducted in January 2024. This article provides detailed information on the beamline optics, the NXE instrument, and its performance and capabilities.

## Abstract 2

The Nanobeam X-ray Experiments (NXE) instrument at the Pohang Accelerator Laboratory X-ray Free Electron Laser (PAL-XFEL) is introduced. The NXE instrument enables users to conduct X-ray experiments with nanofocused X-rays.

## 1. Introduction

The Pohang Accelerator Laboratory X-ray Free Electron Laser (PAL-XFEL) shows outstanding performance in terms of machine stability, particularly regarding beam pointing and arrival time jitter (Kang *et al.*, 2019 ▸). This facility has two serially connected experimental hutches – the X-ray Scattering and Spectroscopy (XSS) hutch and the Nano Crystallography and Coherent Imaging (NCI) hutch – comprising five endstations on a hard X-ray beamline (Eom *et al.*, 2022 ▸). The XSS and NCI hutches are designed to complement each other, thereby enabling users to perform various X-ray experiments. Users can select an appropriate hutch based on their specific experimental purpose by considering the required X-ray techniques and experimental geometry. The XSS is optimized for reflection geometry experiments, including X-ray scattering and X-ray spectroscopy (Kim *et al.*, 2022 ▸; Choi *et al.*, 2023 ▸). It provides micro-focused X-rays with beam sizes of a few tens of micrometres by using a stack of Be compound refractive lenses. The XSS is also equipped with a robotic arm that enables flexible detector positioning. The NCI hutch is dedicated to transmission geometry X-ray experiments, such as coherent diffraction imaging (CDI) and serial-femtosecond crystallography (Nam *et al.*, 2020 ▸; Sung *et al.*, 2021 ▸; Hwang, Kim *et al.*, 2024 ▸; Park *et al.*, 2018 ▸). It utilizes Kirkpatrick–Baez (K–B) mirrors, which enable the X-rays to be focused to approximately 2 µm in both directions (Kim *et al.*, 2018 ▸). Additionally, both hutches are equipped with a femtosecond infrared laser to investigate photo-induced ultrafast phenomena using the optical laser pump–X-ray probe method (Kim *et al.*, 2019 ▸). Since the initiation of user services at PAL-XFEL in 2017, the XSS and the NCI have been continuously developed and optimized, leading to numerous scientific discoveries (Kim *et al.*, 2017 ▸; Assefa *et al.*, 2020 ▸; Hwang *et al.*, 2020 ▸; Kim *et al.*, 2020 ▸; Cho *et al.*, 2021 ▸; Lee *et al.*, 2021 ▸; Kim *et al.*, 2021 ▸; Hwang, Ihm *et al.*, 2024 ▸; Kang *et al.*, 2024 ▸). However, the demand for new experimental apparatus has steadily increased to support emerging scientific research.

Recently, we installed a new instrument at the NCI hutch, specifically designed to provide a nanobeam for X-ray experiments, a capability not available in other instruments at PAL-XFEL. However, other XFEL facilities, such as LCLS (Linac Coherent Light Source), SACLA (SPring-8 Angstrom Compact free electron LAser), European XFEL and Swiss FEL, have already adopted a nanofocusing system. Each of these facilities enables X-rays to be focused to a few hundred nanometres in size using K–B mirrors (Liang *et al.*, 2015 ▸; Mancuso *et al.*, 2019 ▸; Yumoto *et al.*, 2020 ▸). XFEL facilities are continuously striving to improve the flux density of X-rays, paving the way for advancements in a wide range of scientific research fields (Tamasaku *et al.*, 2014 ▸; Fuchs *et al.*, 2015 ▸; Yumoto *et al.*, 2022 ▸; Inoue *et al.*, 2023 ▸). Recently, sub-10 nm focusing optics were introduced at SACLA (Yamada *et al.*, 2024 ▸).

We carefully considered the specifications of the focusing mirrors and highlighted focused beam stability and long working distance as the most critical factors. These considerations were essential for overcoming the challenges associated with having a smaller beam spot. Providing stable user services was the highest priority when designing the K–B mirrors. The long working distance of the K–B mirrors was required to secure enough space for installation of various endstations, allowing for the accommodation of different sample environments and experiment geometries. This flexibility is crucial for conducting diverse X-ray experiments. A Nanobeam X-ray Experiments (NXE) instrument was installed at the most downstream position of the NCI hutch. It comprises an X-ray beam diagnostic system, nanofocusing optics, an X-ray diffraction endstation including a large-area 2D detector, and a femtosecond laser delivery booth. In the following sections, we will describe each component of the instrument in detail. Additionally, we will present the commissioning result to show the performance of the instrument.

## 2. Overview of the NXE instrument

**Figure 1:**
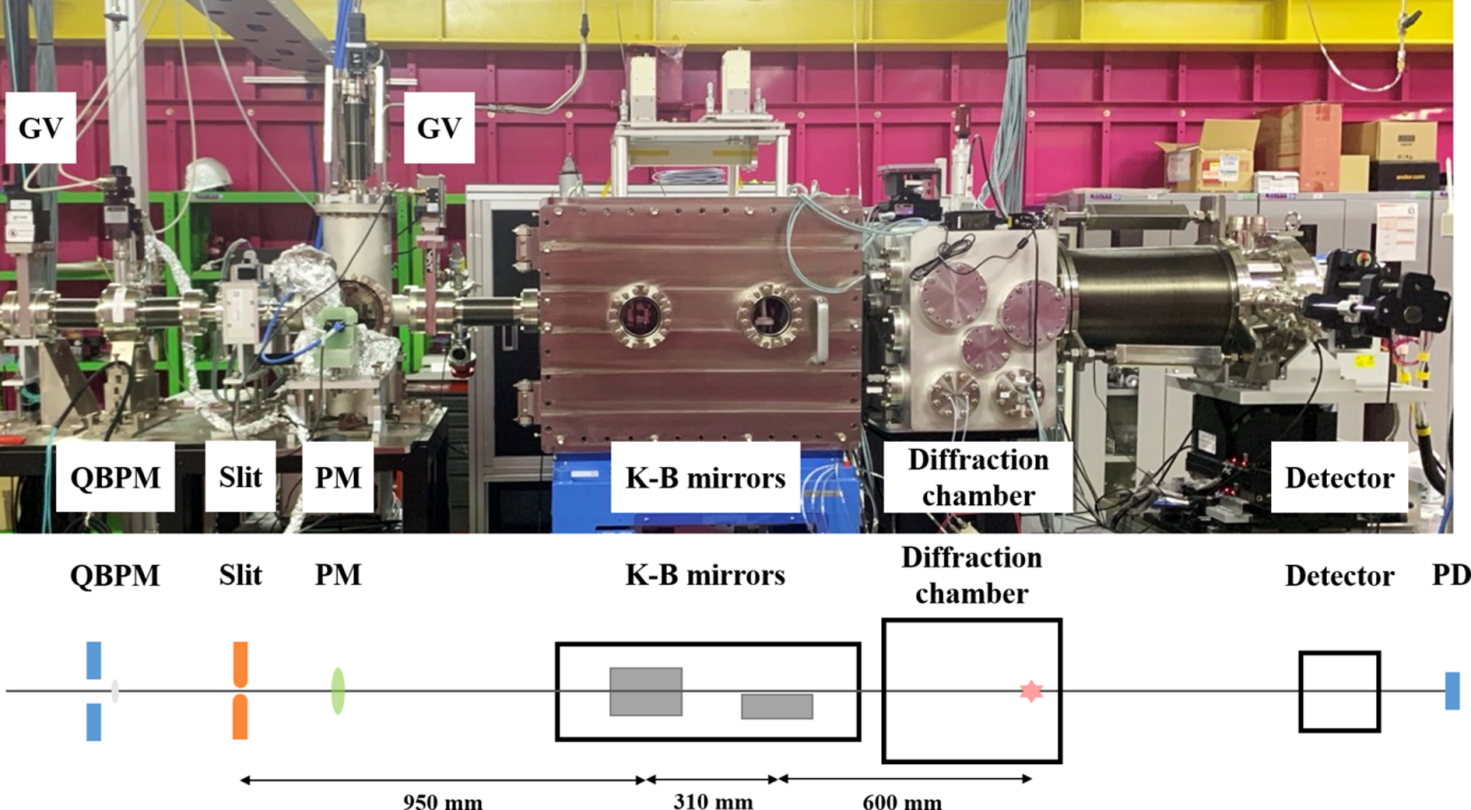
Photograph and layout of the NXE instrument for small-angle X-ray diffraction experiments installed on the Nano Crystallography and Coherent Imaging hutch of the hard X-ray beamline at PAL-XFEL. Key components are labeled: gate valve (GV), quadrant beam position monitor (QBPM), pop-in monitor (PM) and a photodiode (PD).

Fig. 1 ▸ presents a photograph and schematic of the layout of the NXE instrument used for small-angle X-ray diffraction experiments. X-rays are delivered to the NXE instrument after removing high harmonics through double-bounce reflections using a pair of flat mirrors. The beam’s intensity and position are monitored using a diagnostic system, and then focused using K–B mirrors. Two slits located upstream and downstream of the K–B mirrors are used to block unwanted scattering. A chamber is directly connected to the K–B mirror chamber to enable X-ray diffraction experiments to be performed in a vacuum environment. Further, a detector is installed downstream of the chamber through a vacuum beam path. An infrared femtosecond laser is installed to investigate photo-induced phenomena via the optical laser pump–X-ray probe method.

### 2.1. Optical layout

**Figure 2:**
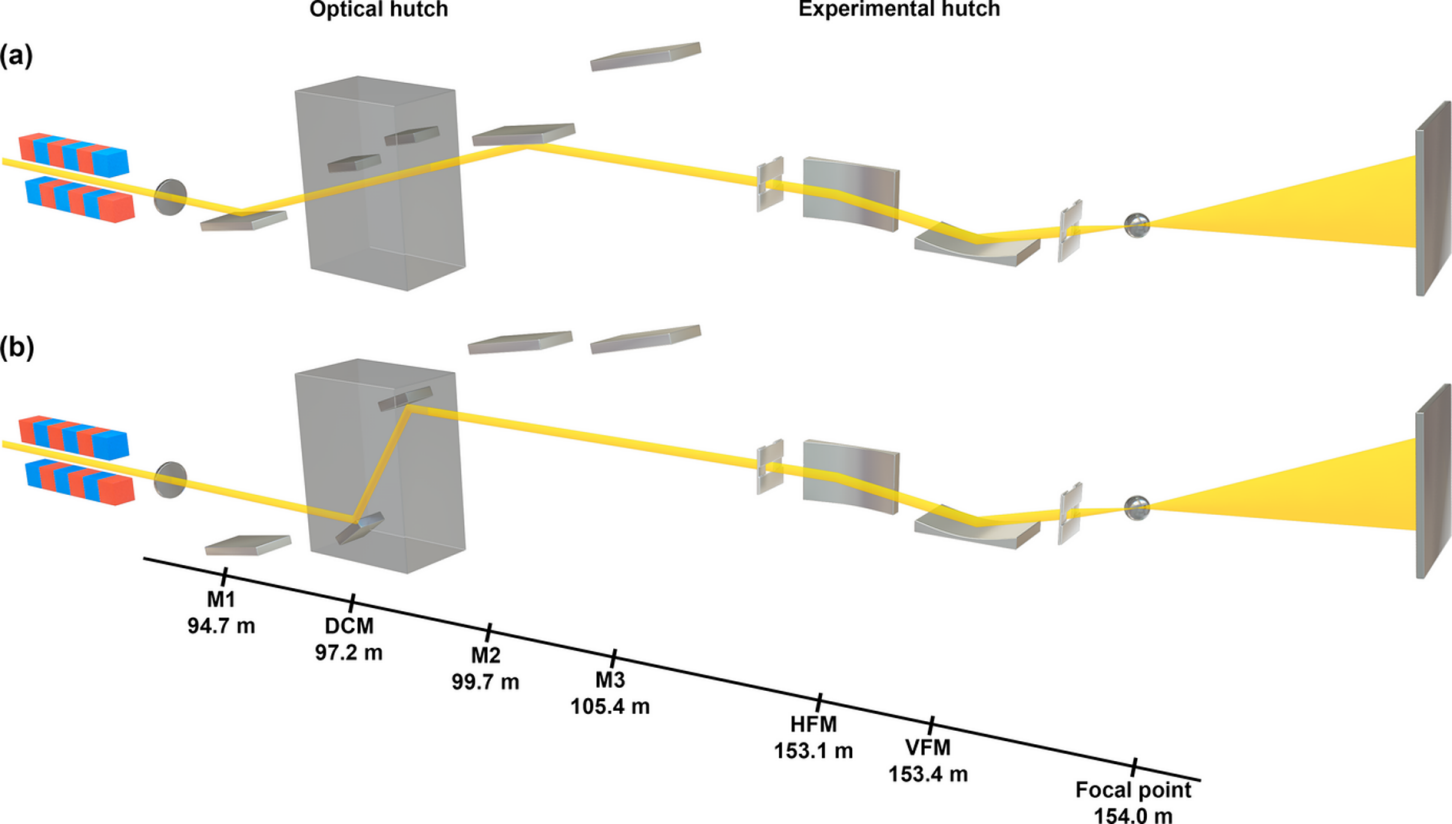
Layout of the optics installed at the hard X-ray beamline of PAL-XFEL. In the optical hutch, three flat mirrors and a Si double-crystal monochromator (DCM) are installed to deliver X-rays to the experimental hutch (EH). Focusing optics at the EH are used to focus the X-rays at the interaction point. (*a*) A pair of mirrors M1–M2 reflects the X-rays with energies lower than 9.5 keV. Due to the cut-off energy, high harmonics are rejected in this configuration. (*b*) The DCM is used alternatively when high-energy resolution is required for X-ray experiments. Key components are labeled: flat mirror (M), double-crystal monochromator (DCM), horizontal focusing mirror (HFM) and vertical focusing mirror (VFM).

The hard X-ray beamline at PAL-XFEL operates in both the self-amplified spontaneous emission (SASE) and self-seeding modes (Kang *et al.*, 2017 ▸; Nam *et al.*, 2021 ▸). In the SASE mode, PAL-XFEL covers X-ray energies ranging from 2.2 to 20 keV, whereas, in the self-seeding mode, X-rays are generated in the range of 3.5–15 keV. Table 1 ▸ shows the key specifications of the beamline. Each operating mode offers unique advantages for conducting X-ray experiments. For a more stable beam with higher flux but relatively broad bandwidth, the SASE beam mode can be used through offset flat mirrors in the optical hutch, as shown in Fig. 2 ▸(*a*). To filter specific photon energies, a Si double-crystal monochromator (DCM) can be employed, although it results in some photon loss, as shown in Fig. 2 ▸(*b*). The self-seeding mode is an alternative, providing a higher flux compared with the monochromatic beam of the SASE mode while maintaining a narrow bandwidth using offset mirrors [Fig. 2 ▸(*a*)]. A new focusing system was designed to produce a smaller and more intense beam than the 2 µm focused beam available in the existing hard X-ray beamline (Kim *et al.*, 2018 ▸). This system was installed at the end of the second experimental hutch to achieve the smallest possible beam size using single-step focusing on the PAL-XFEL beamline (Park *et al.*, 2016 ▸). Fig. 2 ▸ shows the overall layout of the optics in the hard X-ray beamline of PAL-XFEL. Three flat mirrors (M1, M2, M3) are used. The first mirror (M1) is paired with either the second (M2) or the third (M3) to deliver X-rays to the experimental hutch. The M1–M2 pair covers X-rays up to 9.5 keV, whereas the M1–M3 pair delivers X-rays with energies higher than 9.5 keV.

The detailed design parameters of the focusing mirror (JTECH Corporation, Japan) of the NXE instrument are listed in Table 2 ▸. The focusing mirror is 300 mm long and made of synthetic fused silica, with a grazing-incidence angle of 6 mrad and a large spatial capacity of 1.74 mm, which is sufficient to spatially reflect the entire XFEL pulse in both directions. Distances from the expected XFEL source location at the undulator to the centers of the horizontal focusing mirror (HFM) and the vertical focusing mirror (VFM) are 153.125 m and 153.435 m, respectively. The focal lengths of the HFM and VFM are 910 mm and 600 mm, respectively. Assuming the XFEL source size of approximately 50 µm, the focused beam size achieved using the focusing mirror system is approximately 200–300 nm, based on the demagnification ratio. The working distance, set at 450 mm, provides sufficient space for installing a variety of experimental apparatuses for diverse X-ray experiments, while maintaining the desired focused beam size between 200 and 300 nm. The effective area of the focusing mirror is sagittally divided by two optical strip lines, each 7.5 mm wide. Each mirror is divided into two regions: a bare surface to achieve high reflectance at photon energies below 5 keV and an Ir-coated area capable of reflecting up to 12.5 keV.

### 2.2. Diagnostic system

Online photon diagnostics are essential because of the fluctuations of the XFEL beam between pulses. A quadrant beam position monitor (QBPM, FMB Oxford) is used as a representative online diagnostic device at the hard X-ray beamline. The QBPM consists of four photodiodes (PDs) and a thin film in the beam path. The PDs detect backscattered X-rays from the thin film, enabling the determination of the beam position and total pulse energy based on the charges from the PDs (Tono *et al.*, 2011 ▸). The mounted thin films are chemical vapor deposition (CVD) diamond with a thickness of 20 µm and Si_3_N_4_ membranes with thicknesses of 0.5 and 1 µm. The total transmittance of all thin films located in the beam path during the scientific experiments is typically maintained at around 0.9 for the entire photon energy range of the hard X-ray beamline, except in the tender X-ray region. The QBPM is also used to send feedback to the beamline optics, such as flat mirrors or the DCM, to maintain the aligned X-ray position with a resolution better than a few tens of micrometres. In addition to the online diagnostic devices, a pop-in monitor (PM) with a cerimum-doped yttrium aluminium garnet (Ce:YAG) screen is installed in front of the K–B mirror chamber to align the beam and check the beam profile. The screen is positioned perpendicular to the beam and mounted on a holder that can be manipulated as needed. A mirror, angled at 45°, reflects the fluorescent lights from the screen, and the transverse profile of the beam is measured using a charge-coupled device camera.

### 2.3. Endstation for single-shot X-ray diffraction experiments

**Figure 3:**
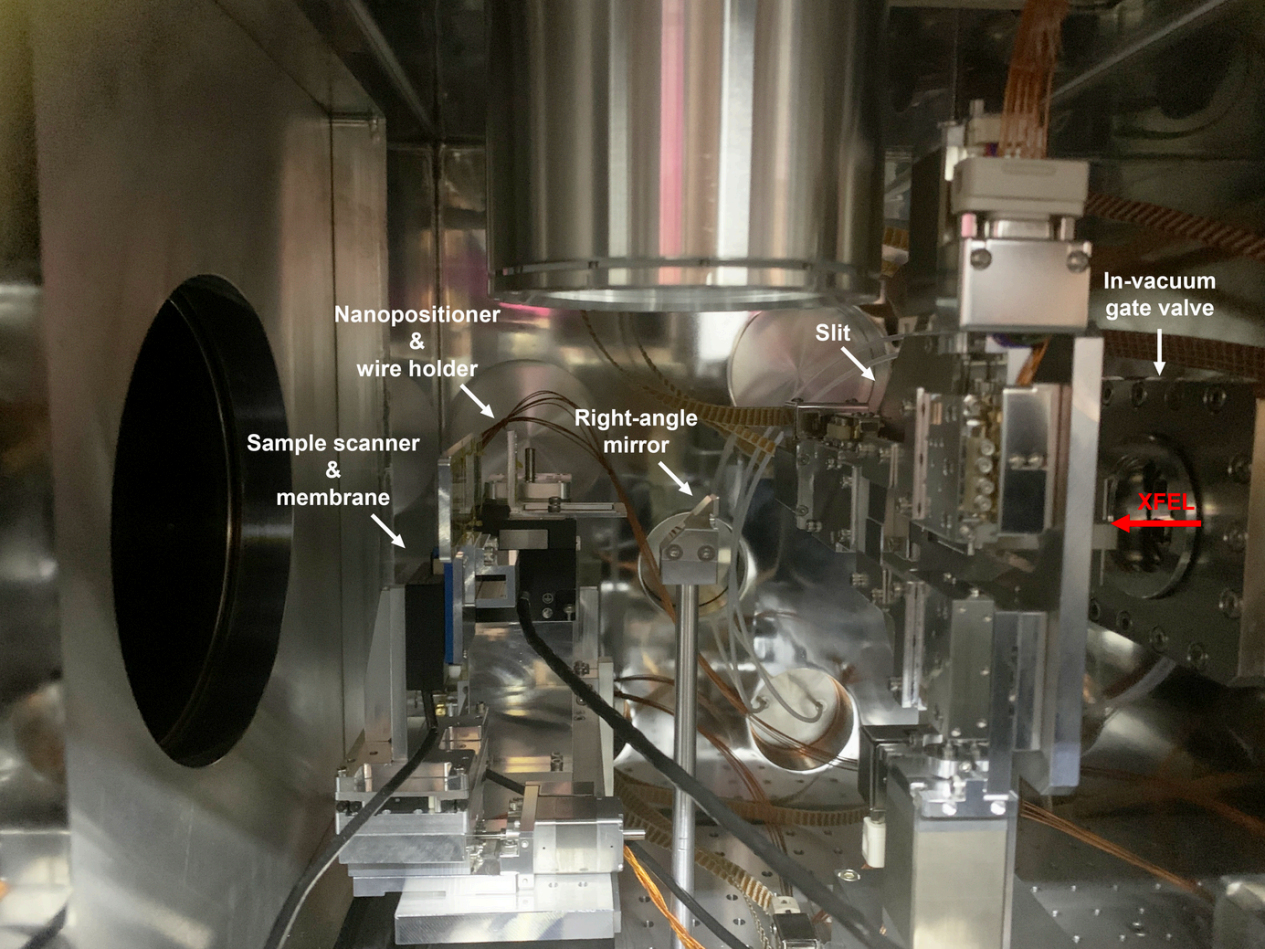
Photograph of an endstation for X-ray diffraction. An in-vacuum gate valve is mounted at the upstream side of the chamber. A four-jaw slit is used for blocking unwanted scattering from the upstream optics. A right-angle mirror, mounted on an optical post, is used for membrane alignment with X-rays. A nanopositioner holding a wire holder and the high-speed piezo stage with three membranes are installed on the *XZ* translation stage assembly.

The first endstation established on the NXE instrument is dedicated to X-ray diffraction experiments conducted in transmission geometry. A vacuum chamber with internal dimensions of 717 mm × 453 mm × 328 mm (W × H × L) is mounted on an *XZ* translation stage and connected to the K–B mirror chamber via the bellows. The overall concept of this chamber is similar to that of the imaging endstation of the coherent X-ray imaging instrument (Sung *et al.*, 2021 ▸). This chamber contains an in-vacuum gate valve, a four-jaw slit stage, a right-angle mirror and a sample stage. Fig. 3 ▸ shows the interior of the chamber.

An in-vacuum gate valve is used to separate the upstream equipment, for example the K–B mirror chamber, when the chamber is opened to exchange the sample. This gate valve is kept open during the experiment. The slit has four 500 µm-thickness silicon blades, each blade mounted on a linear stage. These blades are positioned to block the parasitic scattering from the upstream optics and make a clean beam at the interaction point. A right-angle mirror with a 3 mm-long hole is mounted on an optical post and positioned using a reference laser installed at the beamline. A long-distance microscope is installed above the mirror to observe and align the sample holder with the X-rays. The combination of the microscope and right-angle mirror is used to align the sample holder and synchronize the arrival time of the X-rays and optical laser at the interaction point using Ce:YAG crystals with femtosecond precision timing. The sample stage consists of six motion stages. The two stages in the *X* and *Z* directions are used to position the target with micrometre precision. A compact nanopositioner (P-611.30 NanoCube, PI, Germany) and a high-speed piezo stage are mounted separately on the stack of these two stages. This nanostage can move in sub-nanometre steps and, therefore, it is used to estimate the focused beam size via wire scanning. Fixed-target are used to supply fresh samples. Three motion stages are used to hold the sample holder. For the *X*-direction motion, the high-speed piezo stage is used to implement raster scanning.

### 2.4. X-ray detector

X-ray detectors are crucial for successful X-ray experiments. High sensitivity and a large dynamic range are essential requirements for conducting single-shot experiments using XFELs, which enable the detection of single photons without saturation. To meet these requirements, the adJUstiNg Gain detector FoR the Aramis User station (JUNGFRAU) detector is employed. Two vacuum-operated JUNGFRAU detectors with four-megapixel (4M) and five-megapixel (5M) are available for small-angle X-ray diffraction and wide-angle X-ray diffraction, respectively (Hwang, Kim *et al.*, 2024 ▸).

The 4M and 5M JUNGFRAU detectors are composed of eight and ten modular sensors, respectively. Each sensor has 1024 × 512 pixels with a pixel size of 75 µm × 75 µm. The 4M JUNGFRAU detector features a central aperture of approximately 3.3 mm × 3.3 mm, which is strategically designed to prevent overexposure and damage to the sensor caused by the direct beam. The 5M JUNGFRAU detector has a central hole with a diameter of 15 mm (Hwang, Kim *et al.*, 2024 ▸). The detectors can operate at a frequency of 60 Hz, which corresponds to the maximum operating rate of PAL-XFEL. The specifications of the JUNGFRAU detectors are summarized in Table 3 ▸ and have also been reported previously (Redford *et al.*, 2018 ▸).

### 2.5. Femtosecond optical laser

A femtosecond optical laser is installed at the hard X-ray beamline to investigate photo-induced dynamics with femtosecond temporal resolution. The laser setup comprises a Ti:sapphire oscillator (Vitara-T Coherent Inc., USA), a regenerative amplifier (LegendElite DUO HE, Coherent Inc., USA) and a pulse compressor that can generate femtosecond laser pulses of approximately 40 fs at the fundamental wavelength of 800 nm. The maximum available pulse energy at the interaction point is 5 mJ. Table 4 ▸ summarizes the features of the femtosecond optical laser system of the hard X-ray beamline, PAL-XFEL. When a harmonic generator is used for frequency conversion, the second and third harmonics are generated at 400 nm and 266 nm, respectively, with pulse energies of 1 mJ and 0.7 mJ at the interaction point, respectively. An optical parametric amplifier is also included to provide a broad wavelength range from 240 nm to 2.6 µm, with the maximum pulse energy varying based on the wavelength, *e.g.* 0.3 mJ at 500 nm and 0.5 mJ at 2.0 µm. The details of the laser system have been described elsewhere (Kim *et al.*, 2019 ▸).

## 3. Instrument performance and commissioning result

The NXE instrument is designed to investigate samples using nanofocused X-rays. The nanofocusing effectively enhances the flux density facilitating high-resolution structural investigations of specimens. We present the results of the commissioning experiments conducted to evaluate the performance of the instrument by characterizing the focused beam size and demonstrating single-shot CDI.

### 3.1. X-ray focusing

**Figure 4:**
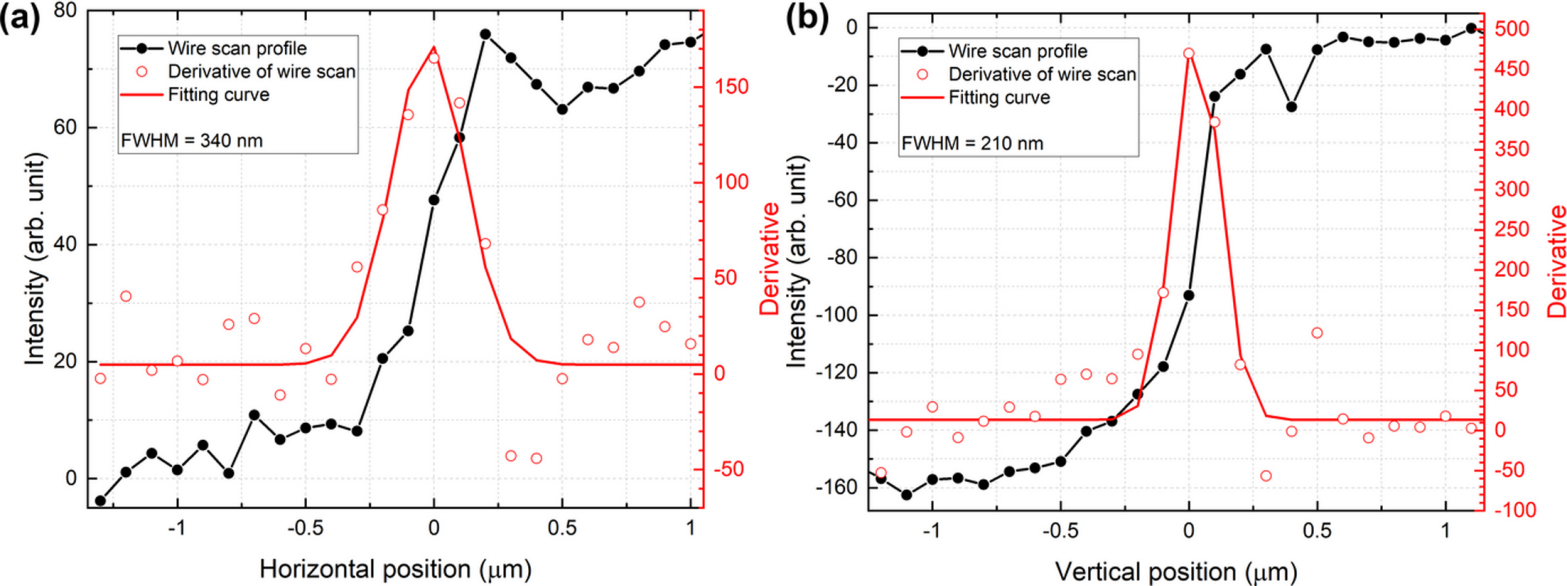
Focused beam size at 9.5 keV X-ray energy was evaluated by the knife-edge scanning method. This implies that the focused beam size is 340 nm × 210 nm (FWHM) in the (*a*) horizontal and (*b*) vertical directions. At each point, more than 30 X-ray pulses were accumulated with a 60 Hz operation mode.

**Figure 5:**
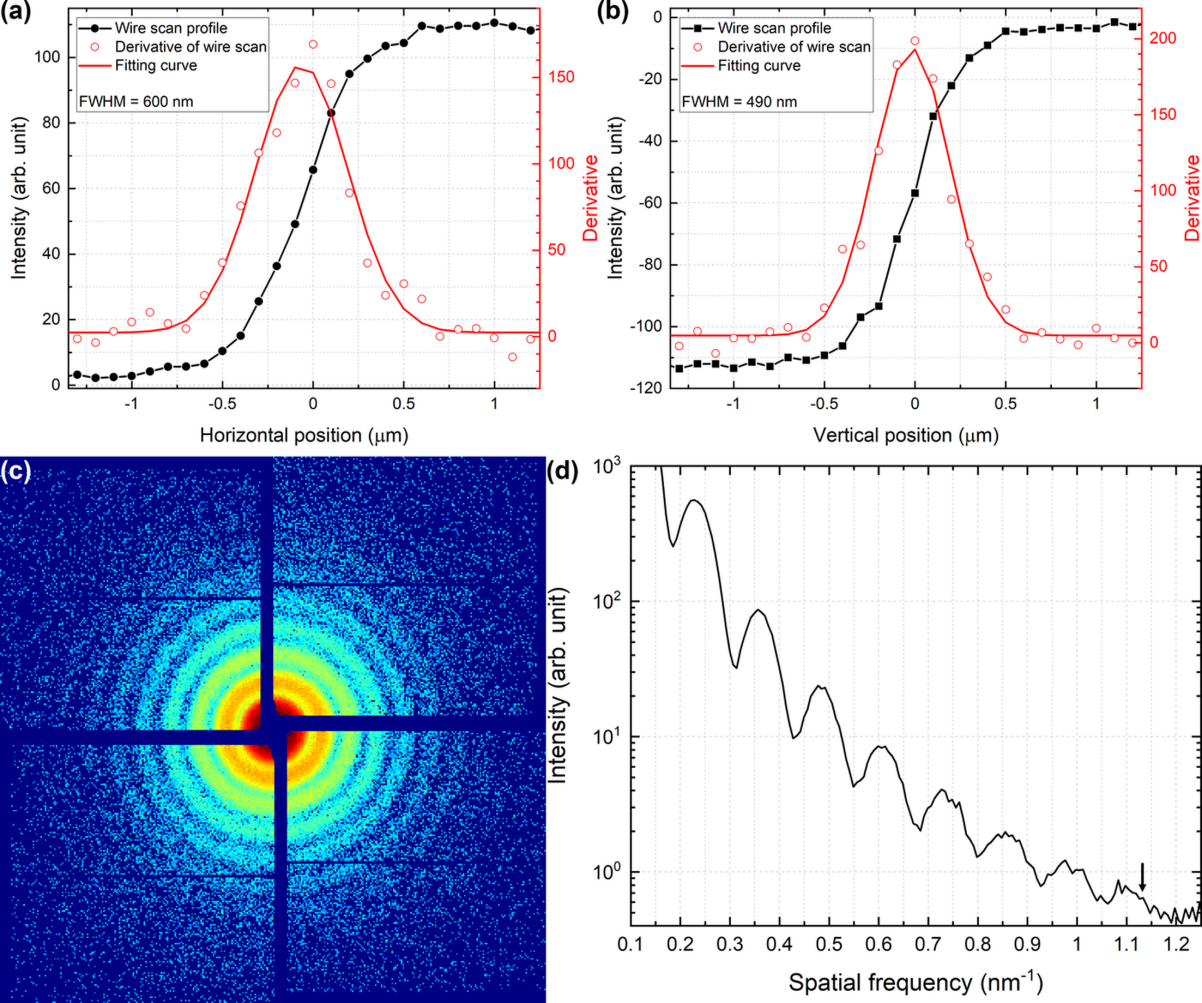
(*a*), (*b*) Focused beam profiles measured by a thin wire. Focused beam sizes in the horizontal and vertical directions are 600 nm × 490 nm in FWHM at an X-ray energy of 5 keV. At each point, 120 X-ray pulses were accumulated with a 60 Hz operation mode. (*c*) Diffraction pattern of a gold nanoparticle with a radius of 50 nm. (*d*) Radial intensity distribution of (*c*). Signals extended to approximately 1.14 nm^−1^, as indicated by a black arrow. This corresponds to approximately 5.5 nm in spatial resolution.

We conducted experiments to characterize the focused beam profile after reflection by two elliptical mirrors that were individually mounted on stacked motion stages with K–B geometry and placed in a vacuum chamber. Before performing the X-ray alignment, rough and perpendicular alignments of the mirrors were completed in air. The incident X-ray energy was fixed at 9.5 keV, which was the highest energy delivered by the M1–M2 pair in the optical hutch. We placed the nanopositioner which can perform sub-nanometre translation and mounted a gold wire with a thickness of 200 µm on the nanostage to measure the focused beam size at the focal plane. The pitch alignment of both mirrors was confirmed via a Foucault knife-edge test using the wire and an indirect X-ray detector (C12849, Hamamatsu Photonics, Japan). We measured the focused beam size using the wire and optimized the pitch position of both mirrors within a few tens of µrad precision. For the measurements in the the horizontal direction, 30 X-ray pulses were accumulated at each scan point using a silicon attenuator with a thickness of 1850 µm. For the vertical direction measurements, 60 X-ray pulses were accumulated at each scan point with a silicon attenuator of 1530 µm thickness. Incident X-rays at 9.5 keV were reduced by at least a factor of 1.67 × 10^−6^ due to the silicon attenuator. Fig. 4 ▸ shows the results of the wire scan in both directions. The magnitude of the standard errors was smaller than the size of the circle symbols at each scan point. The intensity measured by a photodiode gradually decreased after blocking the focused beam using the wire, and the wire entirely blocked the X-rays. The derivative of this scan results in a projected beam profile. The focused beam size was estimated using Gaussian fitting and determined to be 340 nm × 210 nm (H × V) in full width at half-maximum (FWHM). These values were slightly larger than the expected values acquired by the optics simulation. In the simulation to design the dimension of elliptical mirrors, the source size was assumed as 50 µm in both directions. The focused beam size was expected to be 279 nm × 195 nm (H × V, FWHM). For the lower X-ray energy of 5 keV, the focused beam size was 600 nm × 490 nm (H × V, FWHM), which was more than twice as large as the simulated value. In the measurements conducted at 5 keV, 120 X-ray pulses were accumulated at each scan point with a silicon attenuator of 700 µm thickness. Figs. 5 ▸(*a*) and 5 ▸(*b*) show the results of the measurements conducted with 5 keV X-rays. In all the measurements conducted at both X-ray energies, an Ir-coated area of the K–B mirrors was used to focus X-rays at the focal plane and silicon attenuators were employed to protect the wire from the focused X-rays.

### 3.2. Single-shot coherent diffraction imaging

The hard X-ray beamline of PAL-XFEL was equipped with an instrument featuring micro-focusing optics (Kim *et al.*, 2018 ▸). Various X-ray experiments, including CDI experiments, were conducted using this instrument (Assefa *et al.*, 2020 ▸; Hwang *et al.*, 2020 ▸; Cho *et al.*, 2021 ▸; Jung *et al.*, 2021 ▸; Keable *et al.*, 2021 ▸; Kim *et al.*, 2021 ▸). Compared with the micro-focusing instrument, the NXE instrument enables X-ray experiments with a higher flux density. In the CDI experiment, a higher flux density enabled the achievement of a higher image resolution because the image resolution depends on the maximum diffracted angle. The CDI experiment performed using the NXE instrument demonstrated its potential for advanced structural studies.

The incident X-rays were set to 5 keV and the X-ray pulses were focused using K–B mirrors, which resulted in a beam size of 600 nm × 490 nm at the focal plane [Figs. 5 ▸(*a*), 5 ▸(*b*)]. Gold nanospheres with a diameter of 50 nm were prepared for single-shot CDI experiments. Then, the samples were spread onto Si_3_N_4_ membranes. As the specimens were destroyed by each focused X-ray pulse, fresh specimens were supplied for each incoming X-ray pulse. The fresh samples were supplied using the fast piezo stage. A total of 271 X-ray pulses arrived at the interaction point when the stage moved by 9 mm at 540 mm s^−1^. A total of 271 diffraction patterns were collected using the 4M JUNGFRAU detector during every run, with the detector positioned 800 mm from the interaction point. A representative diffraction pattern with a binning of 3 × 3 is displayed in Fig. 5 ▸(*c*). The radial summation of the diffraction pattern is shown in Fig. 5 ▸(*d*). The signal extended to 1.14 nm^−1^, which corresponds to approximately 5.5 nm in the spatial resolution.

Additionally the flux density of the nanofocused beam was estimated by fitting diffraction patterns from a single gold nanosphere. This revealed a maximum flux density of 3.9 × 10^11^ photons µm^−2^. Compared with PAL-XFEL’s micro-focusing beam, this represents an enhancement of approximately 27.8 times (Lee *et al.*, 2020 ▸).

## 4. Conclusion

The NXE instrument was installed at the hard X-ray beamline of PAL-XFEL. This is the first instrument at PAL-XFEL to employ nanofocusing optics, which significantly enhances the flux density of the focused beam, achieving approximately 27.8 times the flux density of a micro-focusing beam. We conducted performance tests to confirm the focused beam size at two photon energies of 5 keV and 9.5 keV. At these X-ray energies, the focused beam sizes were 600 nm × 490 nm and 340 nm × 210 nm, respectively. In addition, we successfully demonstrated single-shot CDI experiments using nanobeams. The signals of the single-shot diffraction pattern from the gold nanosphere with a diameter of 50 nm extended to 1.14 nm^−1^ with a good signal-to-noise ratio. In 2023, the NXE instrument was opened to PAL-XFEL users to conduct X-ray experiments. Furthermore, we plan on developing a new endstation that will enable X-ray scattering and X-ray spectroscopy experiments to be conducted on liquid-phase specimens. The next step in this plan is to provide a variety of sample environments for X-ray experiments.

## Figures and Tables

**Table 1:** Key specifications of the hard X-ray beamline at PAL-XFEL

	Beam mode		
	SASE		Self-seeding
	Natural bandwidth	Si(111) DCM	
Photon energy	2.2–20 keV		3.5–15 keV
Bandwidth	∼0.3%	∼0.01%	∼0.005%
Pulse energy (at 9.7 keV)	∼2 mJ	∼0.06 mJ	∼1 mJ
Repetition rate		60 Hz	
Pulse duration		∼40 fs (FWHM)	

**Table 2:** Optical parameters of the focusing mirrors (RMS = root mean square)

	Horizontal focusing mirror	Vertical focusing mirror
Surface profile	Tangential ellipse	
Substrate material	Synthetic fused silica	
Surface coating	Iridium 50 mm (7.5 mm width), non-coated (7.5 mm width)	
Mirror substrate size	300 mm × 50 mm × 50 mm (L × W × H)	
Clear aperture	290.3 mm × 15.05 mm (L × W)	290.7 mm × 15.05 mm (L × W)
Tangential slope error	0.065 µrad (RMS)	0.066 µrad (RMS)
Sagittal slope error	0.069 µrad (RMS)	0.296 µrad (RMS)
Roughness	0.124 mm (RMS)	0.089 mm (RMS)
Grazing-incidence angle	6 mrad	
Source distance	153.125 m	153.435 m
Focal length	910 mm	600 mm
Spatial acceptance	1.742 mm	1.744 mm

**Table 3:** Summary of the main specifications and features of JUNGFRAU detectors at PAL-XFEL

Module sensor specifications and features		
Silicon sensor	Thickness	320 µm
	Pixel size	75 µm × 75 µm
	No. of pixels	512 × 1024
ASIC chip	No. of ASIC/sensors	8 (2 × 4 formation)
	No. of pixels	256 × 256
Sensing area	4 cm × 8 cm	
Detectable energy range	>∼2 keV at single photon sensitivity	
Dynamic range	∼10^4^ at 12 keV photons^−1^ pulse^−1^ pixel^−1^	
Frame rate	<1.2 kHz continuous	

4M JUNGRAU detector		
No. of modules	8 (2 × 4 formation)	
Detection area	16 cm × 16 cm	
Size of central aperture	3.3 mm × 3.3 mm	

5M JUNGFRAU detector		
No. of modules	10 (2 × 5 formation)	
Detection area including central vacancy	20 cm × 20 cm	
Size of central vacancy	5 cm × 12 cm	
Size of central hole	15 mm	

**Table 4:** Summary of the femtosecond optical laser

Ti:sapphire laser system	
Wavelength	800 nm
Repetition rate	120 Hz
Pulse energy (at the interaction point)	5 mJ
Pulse duration	∼40 fs

Frequency tuning laser system	
Harmonic generator	400 nm and 266 nm
Optical parameter amplifier	240–2600 nm
